# Evolving SPECT-CT technology

**DOI:** 10.1093/bjr/tqae200

**Published:** 2024-10-09

**Authors:** Kathy P Willowson, Dale L Bailey

**Affiliations:** Department of Nuclear Medicine, Royal North Shore Hospital, Sydney, NSW 2065, Australia; Institute of Medical Physics, University of Sydney, Sydney, NSW 2065, Australia; Department of Nuclear Medicine, Royal North Shore Hospital, Sydney, NSW 2065, Australia; Faculty of Medicine and Health, University of Sydney, Sydney, NSW 2065, Australia

**Keywords:** SPECT, CT, imaging technology

## Abstract

Both hardware and software developments have seen single photon emission CT (SPECT)/X-ray CT technology grow at a rapid rate. Such growth has been fuelled by the need for clinical applications and has provided inspiration for clinical developments, particularly with the expanding role of theranostics. Developments such as whole-body quantitative reconstructions, digital detectors, and recent multidetector 3D geometry have allowed SPECT to become comparable to PET on a number of fronts, with a particularly powerful role in biodistribution and dosimetry studies for both planning and evaluating radionuclide therapy. Whilst there remain fundamental challenges for SPECT such the limited spatial resolution and sensitivity, the unique opportunity to image long-lived radioisotopes and simultaneous multi-tracer studies, together with easily accessible equipment, makes SPECT/CT a valuable clinical asset. This review discusses developments in SPECT/CT technology and their clinical impact.

## Introduction

Hybrid single photon emission CT/X-ray CT (SPECT/CT) scanners first became clinically available in 1999 with the introduction of the HawkEye system (GE HealthCare, Milwaukee, United States) equipped with a low-resolution single-slice CT.^1^ This was followed closely in 2004 with the release of the Symbia T2 (Siemens Healthineers, Erlangen, Germany) and Precedence (Philips HealthCare, Milpitas, CA, United States) systems equipped with fully diagnostic CT components. In the 20 years since its initial introduction, clinical SPECT/CT has seen some major developments in technology, many of which have been fuelled by the unique capabilities and ready access to SPECT systems around the world.

When compared to positron emission tomography (PET), SPECT has some inherent advantages in addition to ease of access. This includes the ability for dual nuclide acquisitions, imaging radionuclides that are typically associated with a longer half-life, and imaging gamma emissions that accompany many therapeutic beta-emitters following radionuclide therapy. Thus, SPECT lends itself very readily to not only a large suite of diagnostic studies but also theranostic applications, which has been a major driver behind many of the developments in recent years.

The addition of fused CT data was originally aimed at providing a low-dose option for CT-based attenuation correction with the additional benefits of accurate anatomical localisation when reviewing SPECT images.^2^^,^^3^ The role of CT has since expanded, and such diagnostic quality CT data can now be used for guiding image reconstruction, accurate anatomical auto-segmentation, and being incorporated into further photon transport corrections for accurate SPECT quantification.

This review highlights the recent developments in SPECT/CT technology including both the acquisition hardware and reconstruction software, as well as further developments that may have a clinical impact together with future challenges.

## Acquisition hardware and geometry

Historically, gamma camera hardware has remained largely unchanged since the introduction of the Anger camera in 1958.^4^ Such a design is built upon a solid NaI(Tl) crystal of thickness 9 or 16 mm, depending on the intended gamma energies to be imaged. The thicker the crystal, the higher the sensitivity yet the poorer the intrinsic spatial resolution, although on modern systems this degradation is minimal and virtually negligible when compared to the spatial resolution of the collimator. Photons that pass through the lead collimator attached to the front of the crystal will be absorbed by the crystal leading to the emission of visible light photons which are detected by an array of photomultiplier tubes (PMTs) fitted directly to the back of the crystal. The light photons detected by each PMT are converted to an electrical signal which is evaluated to determine the position and energy of the event before contributing to the image matrix. Most current generation SPECT systems consist of two detectors that can be arranged in various configurations around the patient, however single and triple head systems have also been prominent. Figure 1 represents a typical SPECT/CT set-up.

**Figure:**
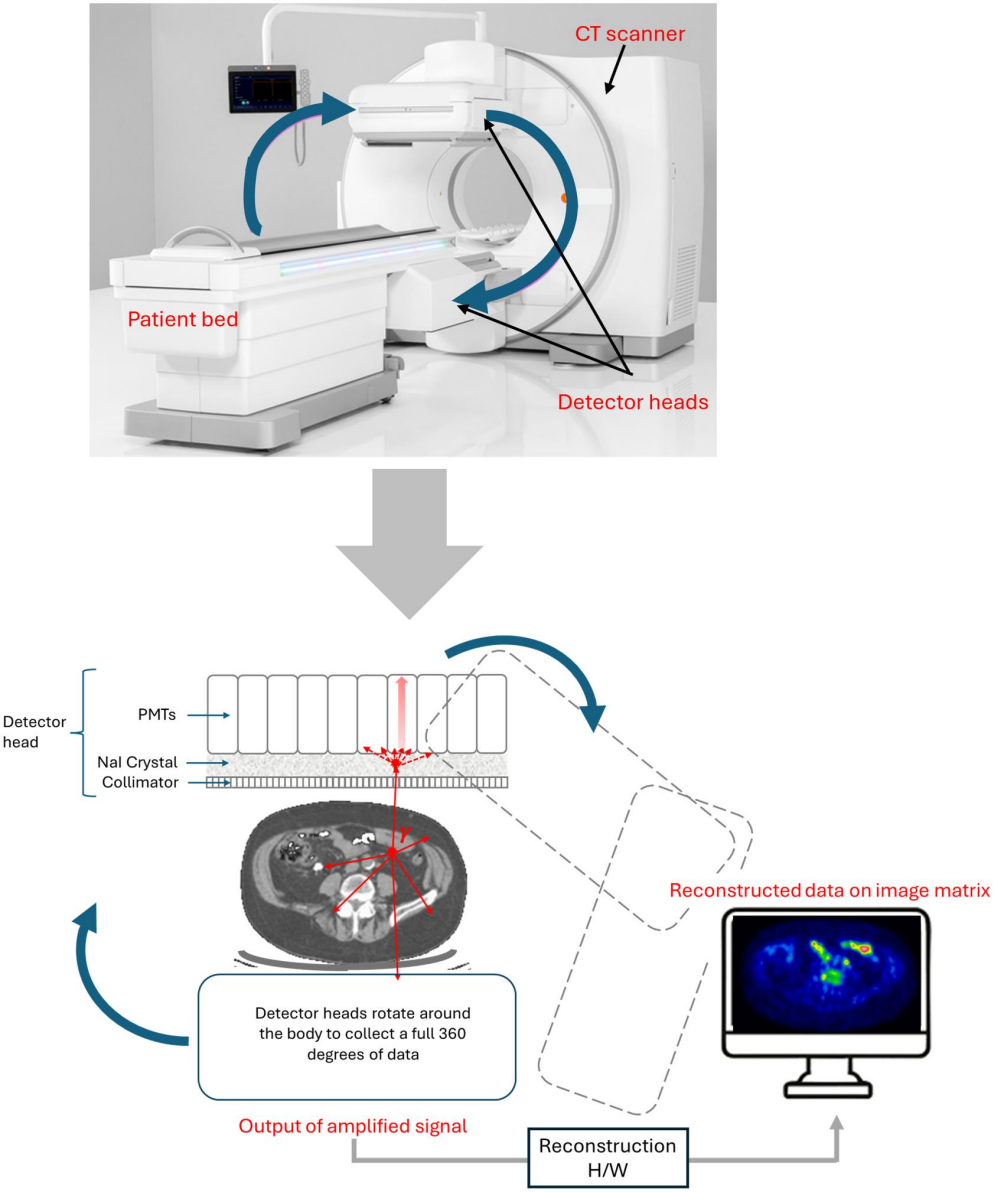
Figure 1. Representation of traditional NaI based SPECT/CT hardware and the acquisition process.

### Detectors

A major area of development in current state-of-the-art SPECT systems is the detector material. Whilst NaI(Tl) is a cost-effective option that is readily available with many favourable characteristics, the lengthy process of detection and conversion *via* PMTs leads to a large uncertainty in measured photon energy [typically ∼9-10% full width at half maximum (FWHM) for 140 keV photons^5^]. This ultimately leads to poor energy resolution combined with a limited intrinsic spatial resolution that deteriorates that must be balanced against any increase in system sensitivity. In a bid to improve this design, recent systems have introduced solid state detectors based on the material cadmium-zinc-teluride (CZT).

Whilst CZT detectors are more expensive than using NaI(Tl) scintillation detectors, there are many advantages to such a design. CZT acts as a semiconductor, directly converting the detected gamma photons into an electrical signal without the need for bulky PMTs (Figure 2). Such a process involves ionizations in the CZT material due to both photoelectric and Compton interactions which act to create an induced charge. By applying a high voltage between the front and back surface of the detector, the front behaves as a cathode whilst the pixelated back surface acts as an array of individual anodes, which collect the induced charges. Overall, the design acts to improve the spatial resolution (in the order of ∼2.4 mm^6^) and energy resolution (typically 2%-5% for 140 keV photons^6^) with the additional advantage of a compact and flexible design that also improves system sensitivity. Furthermore, dead time and saturation are not an issue in high count studies (such as post-therapy) due to the fact that each pixel acts as a separate detector, improving quantitative accuracy. The CZT material also offers the ability to have a flexible design including closer positioning of the detector to the patient and has the further advantage of a larger effective field-of-view (FoV) due to the lack of distortion that occurs with edge effects in NaI(Tl)/PMT designs.

**Figure:**
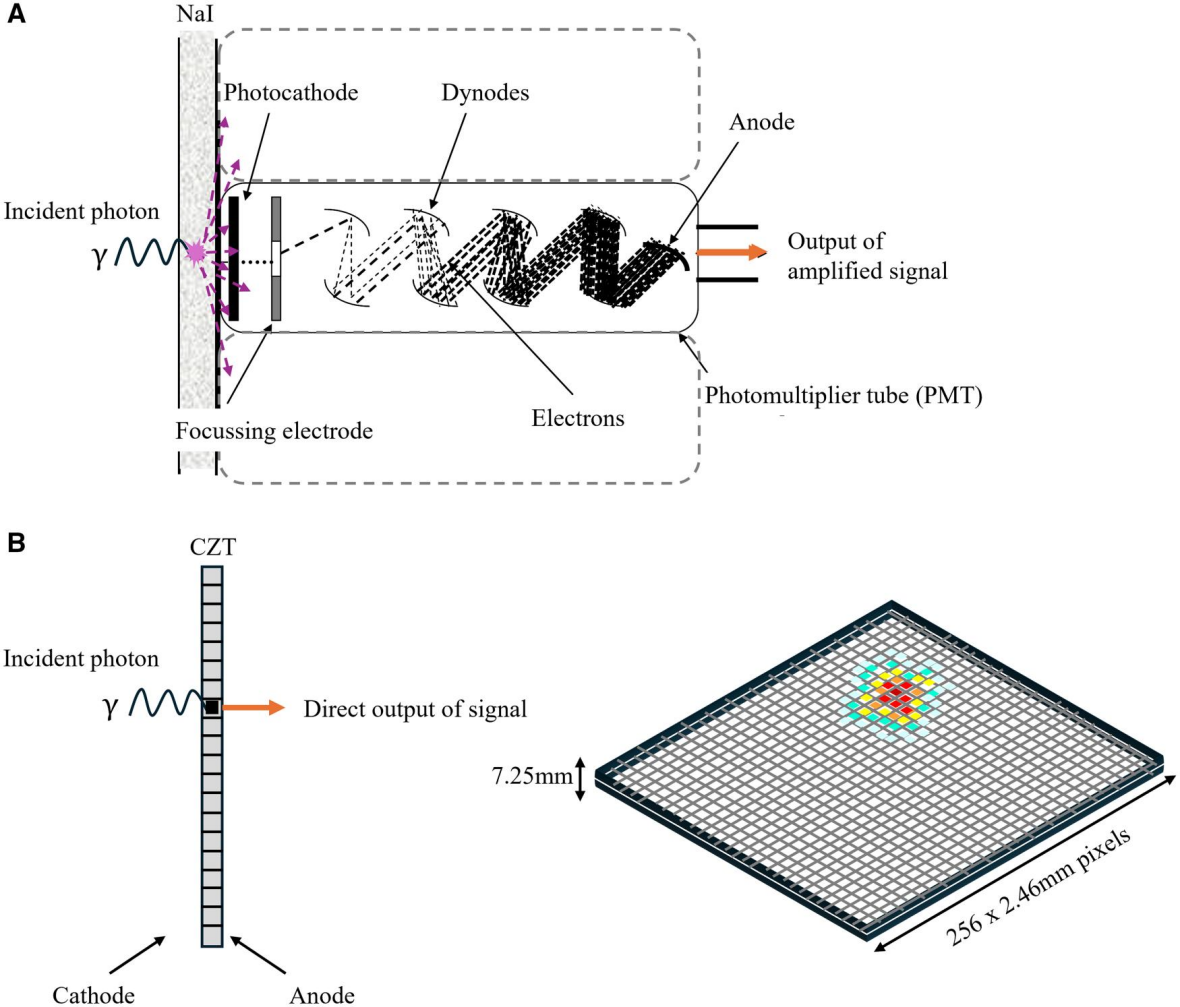
Figure 2. Comparison of the conventional gamma camera design with PMTs (A) vs the considerably 1 less bulky digital semiconductor design with CZT (B).

Table 1 includes examples of SPECT detector and system design properties, which are discussed in detail below, in comparison to PET.

**Table 1.** Comparison of common properties for conventional, digital, and multidetector-3D (ring geometry) SPECT systems.

Parameter	PET^a^—standard	PET^a^—total body	SPECT^b^—conventional	SPECT^b^—CZT	SPECT^b^—multidetector 3D
Spatial resolution (FWHM)	∼3.2 mm	∼3.2 mm	∼22 mm (MEGP)^54^	10.4 mm (MEGP)^c^	≤10 mm (TBC)
System sensitivity (cps/MBq)	16 000 (∼800×)	176 000 (∼9000×)	19.5 (1.0×)^55^	15 (∼0.75×)	42 (∼2.0×)^d^
“Whole body” image acquisition time	∼15 min	3-5 min	30-40 min	20 min	12-15 min
Quantitative accuracy (typical limits)	±2.5%	±2.5%	±10%	±5%	±5%
RC for 17 mm sphere in NEMA NU-2 IQ phantom	1.0	1.0	0.18	0.34^29^	0.42

a For ^18^F.

b All SPECT figures quoted for combined 113 & 208 keV peaks of ^177^Lu.

c GE HealthCare NM/CT 870 CZT dual head system.

d Spectrum dynamics value.

### Collimators

Traditionally the collimator has been the major limitation to SPECT sensitivity, allowing less than 1 of every 5000 gamma rays to be detected in a standard ^99m^Tc image,^6^ whilst also contributing to the determination of achievable spatial resolution. The collimator is required to allow only those photons travelling perpendicular to the face of the detector to be detected and hence contribute to correct positional information in the image. Some sophisticated designs in collimation have been introduced, particularly in dedicated cardiac imaging, such as the SmartZoom system (Siemens Healthineers, Erlangen, Germany) which allows the central part of the collimator to focus directly on the heart providing magnification and an increase (up to four times) in the sensitivity over this region. In general, any increase in the projected image size results in a sensitivity improvement as well, as more photons from the region of interest are detected. Other vendors have tweaked the traditional lead septa, such as GE HealthCare’s low energy high resolution and sensitivity (LEHRS) collimator, which uses slightly shorter and thinner septa to allow higher sensitivity without a loss in resolution (92 cps/MBq vs 72 cps/MBq on the traditional low energy high resolution collimator).

The introduction of CZT systems has seen “matched” collimators with square holes and septa that are directly registered to gaps between the anodes on the back end of the semiconductor detector. This acts to fix the pixel size of the image and reduce the intrinsic spatial resolution of the system with better event localisation (GE quote a FWHM of 2.8 mm compared to 4.3 mm for a standard NaI(Tl) system). The drawback, however, is that the collimator design is not independent of the detector and thus it cannot lend itself to imaging all radionuclide energies. A major shift in traditional design has also been realised in the state-of-the-art CZT systems with a ring-based geometry (see below), which are fitted with tungsten collimators in place of lead due to its higher density (19.3 g/mL) and hence stopping power for energetic gamma photons. It is reported that tungsten collimators provide superior spatial resolution and improved image contrast when imaging ^131^I, ^177^Lu, and ^123^I^7^ due to thinner septa, with an additional advantage of being compatible with MR hybrid system design.

Novel approaches to collimator design and manufacture have also been explored utilising new technology, such as 3D printing, to allow for precision and flexibility that is not available through other traditional means. A full review of both traditional and state-of-the-art collimator design and construction can be found in Van Audenhaege’s review.^8^

### Orbit

Current generation SPECT scanners are no longer limited to a circular orbit but will vary the distance at which each projection is acquired in a patient-contour orbit. Such projection data can typically be acquired in either traditional “step and shoot” mode or “continuous” mode, where the detector head is not required to physically stop at each projection angle but instead moves incrementally throughout the acquisition. The premise behind such technology is of course to allow the detectors to be in close proximity to the source, improving the overall spatial resolution which is largely governed by the collimator and which deteriorates rapidly with distance from the emitting source. The Siemens SmartZoom has a cardio-centric orbit to ensure the organ of interest remains at the centre of the acquisition arc and hence in the area of greatest magnification.

GE HealthCare CZT systems are also equipped with SwiftScan technology, allowing the scanner to continue acquiring data when moving between projections steps. The data acquired during this movement is apportioned to the closest acquisition projection angle (ie the first half of the data will be added to the previous projection and the second half of the data added to the following projection). As such it differs to the standard continuous mode of acquisition for typical SPECT scans which acquire continuously through the entire rotation of detectors thus suffering from resolution losses. This technology reportedly leads to higher counts and a higher contrast-to-noise ratio in reconstructed data, which together with the vendor’s new collimator design, is claimed to allow for a reduction in either patient dose or scan times by up to 25%.

### Geometry

One of the most radical changes to SPECT/CT technology has been realised with fixed ring geometry scanners, or multidetector 3D designs, which have only been made possible with the introduction of CZT detectors. Two such systems are currently on the market with a similar design, GE HealthCare StarGuide and Spectrum Dynamics Veriton systems. Figure 3 demonstrates the design principles. Such SPECT/CT systems follow the fundamental acquisition principles of the original dedicated cardiac systems of a similar nature (D-SPECT, Spectrum Dynamics, Caesarea, Israel and Discovery NM 530c, GE Healthcare, Haifa, Israel), that were first introduced with a fixed C-shaped geometry employing the swivelling CZT detector technology.

**Figure:**
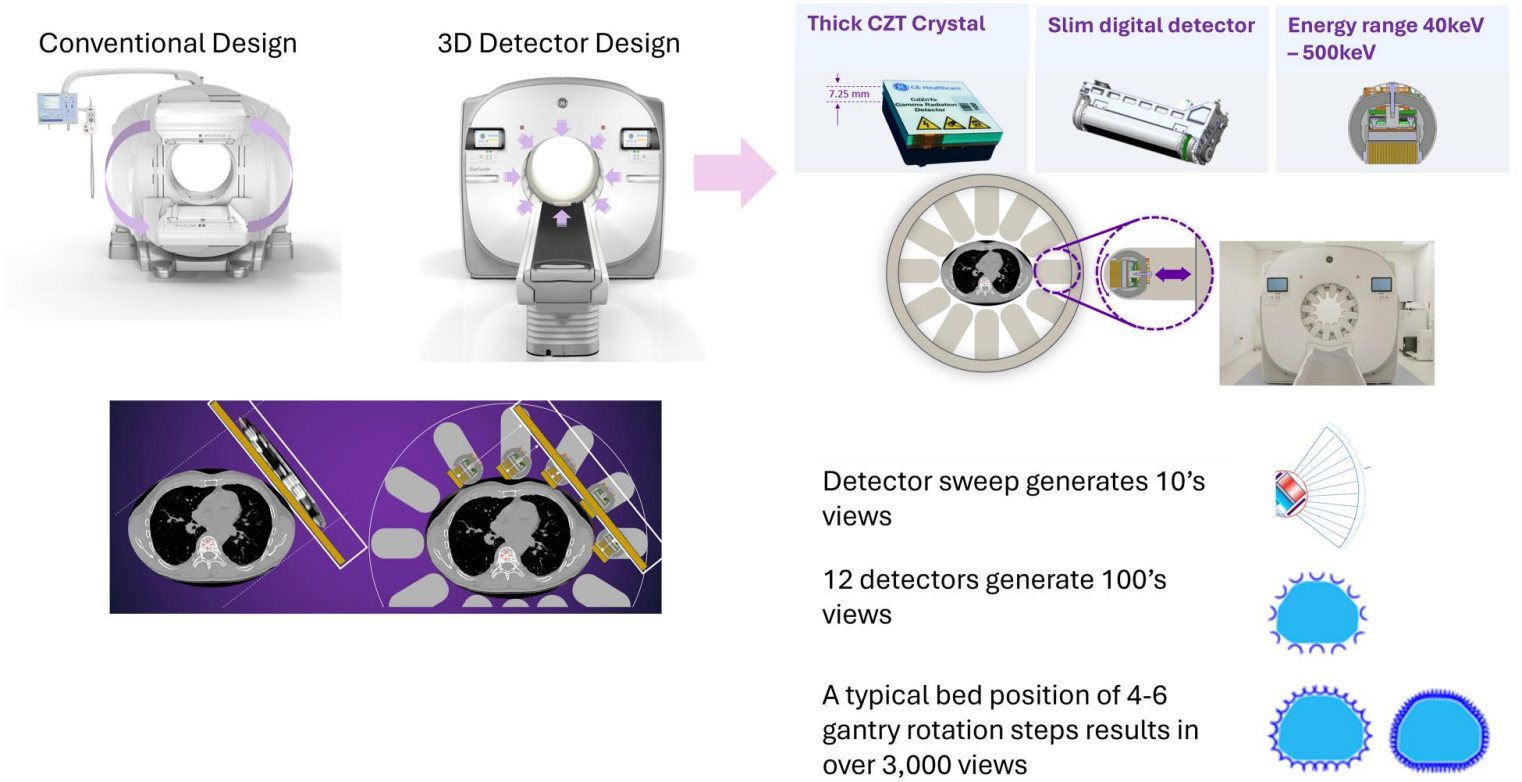
Figure 3. Example of conventional SPECT gantry design versus the new ring geometry (or 1 multidetector 3D) design. Images courtesy of GE HealthCare.

The Veriton system makes use of 12 individual digital detector arms (4 × 32 cm in size) arranged in a full circle around the patient, attached to the 80 cm bore and coupled to a standard diagnostic CT. Each of these detectors can independently move to achieve adaptive body contouring and minimise distance from the surface of the patient, thus improve resolution further. Each of the CZT columns swivels during acquisition resulting in a full 360 degrees acquisition when data from all detectors is combined with a design that allows high sensitivity and rapid scanning. The technology also lends itself to dynamic SPECT acquisition, which is an area of evolution. The development of digital detectors and improvements in scanner sensitivity may see this change, with particular interest already demonstrated in coronary artery disease^9^ and liver function.^10^

There is the disadvantage that these systems do not allow for collimator exchange and thus have been designed with certain radionuclides in mind, particularly for theranostic imaging of ^177^Lu where the improved energy resolution allows both photopeaks (113 and 208 keV) to be acquired. However, use with standard diagnostics such as ^99m^Tc is still possible.

Further comparisons with standard geometry scanners can be seen in Table 1.

## Software and reconstruction

### Reconstruction algorithms

Statistical iterative methods have been the gold standard in SPECT reconstruction for many years, and remain a powerful tool given the ability to incorporate additional physical information into the reconstruction process. Iterative reconstruction does not suffer from many of the noise and image artefacts introduced with back projection techniques, and current computing power and accelerated algorithms mean it is a fast option for the generation of clinical data. Whilst the traditional iterative algorithms, such as the maximum likelihood expectation maximization (MLEM) algorithm, incorporate all projection data into each iteration, accelerated algorithms such as the ordered subset expectation maximization (OSEM) algorithm^11^ base each iteration on only a nominated subset of the available projection data.

The accelerated process gives the user the option to modify the number of iterations and subsets which ultimately strikes a balance between convergence or image accuracy and image noise which increases with iteration number. Hence data can suffer from either excessive noise or alternatively blurring due to the need for post-reconstruction smoothing to improve the signal-to-noise ratio. An additional criterion relating to limitations on image noise can be incorporated using the introduction of prior knowledge as a constraint. In this case, noisy images are penalised through methods such as the comparison of reconstructed pixels to their neighbours, which in noise-free data should be reasonably consistent. The neighbourhood method must of course account for edges, where a high difference is to be expected, and thus a threshold on difference is usually set to avoid edge blurring. Alternatively, anatomical priors can be used to only compare pixels within common anatomical structures. Such algorithms often utilise Bayesian methods or *Maximum A Posteriori* (MAP) algorithms.^12^^,^^13^

Anatomically guided priors from the associated hybrid CT component can be used for Bayesian methods and have been successfully incorporated into the reconstruction of SPECT bone data.^14^ Superior quantitative accuracy has been demonstrated when compared to standard OSEM, and such an approach has been incorporated into the Siemens’ xSPECT Bone reconstruction algorithm.^15^ A variant is the ordered subset conjugate gradient algorithm (OSCGM), such as the Siemens’ xQUANT algorithm,^16^ which allows for an improvement in image resolution and quantification through the use of differing noise and convergence properties when compared to standard OSEM^17^ (Figure 4).

**Figure:**
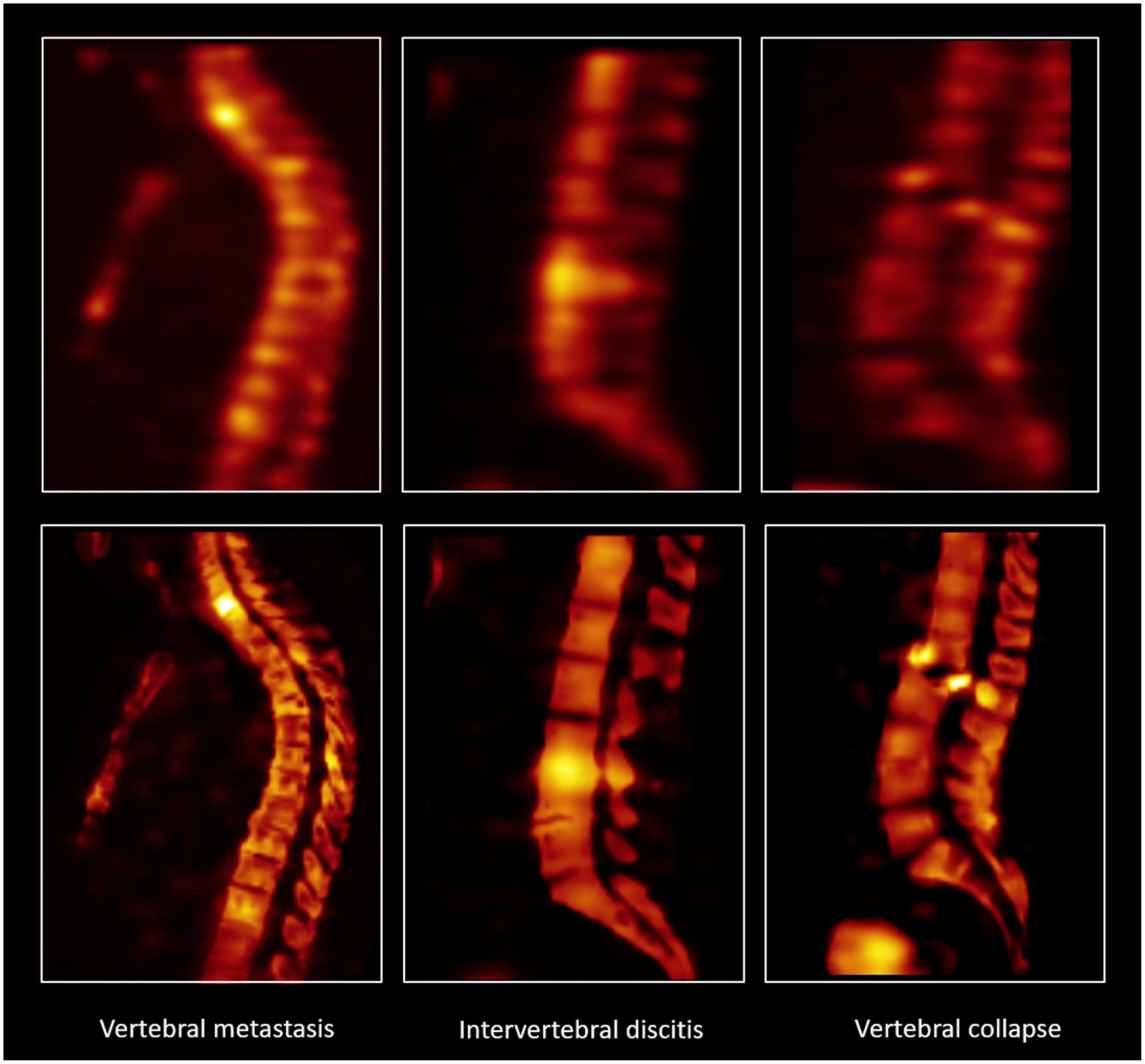
Figure 4. Example of OSCGM reconstruction with Siemens’ xSPECT bone algorithm compared to a standard bone scintigraphy reconstruction, demonstrating improved delineation of vertebral structure. Image provided by Siemens Healthineers, data courtesy of John Hopkins University, Baltimore, MD, USA Reference S119_22 (left), the University of Erlangen, Germany, Reference FAU_VS36 (middle) and Friedrich Alexander University, Erlangen, Germany Reference S157_21 (right).

With an increase in computing power, complete photon transport modelling has become a reality through Monte Carlo based reconstruction algorithms. The simulation of all relevant physical effects can achieve inherently quantitative data with high accuracy. Whilst accurate transport modelling for reconstruction was originally taking in the order of 30 min,^18^ huge improvements have been realised with reports of ^177^Lu image reconstruction in as little as 3 min or less.^19^ There has also been particular interest in accurate bremsstrahlung SPECT reconstruction of ^90^Y^20^ and full energy spectrum reconstruction of ^166^Ho^21^ for post-raidoembolization dosimetry, and more recent exploration into the radionuclide ^111^In^22^ which traditionally suffers from exceptionally poor spatial resolution.

### Acquisition mode

SPECT data are traditionally stored as binned data, where collected photons are saved based on position and projection angle. An alternative approach to data storage in SPECT is that of list mode, where qualities of detected photons including the energy absorbed, time of detection are estimated and stored in a list format allowing additional information to be preserved and manipulated in post-processing with greater precision. Such methods are on par with PET data storage and can improve performance and quantification when compared to standard binning or static acquisition techniques.^23^ List mode acquisition is now available from many of the major vendors and gives more flexibility to post-processing, allowing generation of static, gated or dynamic data albeit at the cost of large storage requirements. List mode acquisition also allows for data to be acquired during detector motion to improve sensitivity and reduce image noise,^24^ a quality known as “fast SPECT” which is currently being exploited in the new GE HealthCare SwiftScan technology. It also has benefits for low count multi-energy window studies, such as for ^67^Ga imaging, in terms of quantification accuracy of reconstructed images.^25^

### Quantification

Quantification in SPECT refers to reconstructed data in units of absolute radioactivity, or commonly *kBq/cc*. Such data add additional information to what has traditionally been considered as a qualitative imaging modality only. There are several corrections that must be performed to convert detected photons into units of absolute radioactivity. These include photon attenuation, the degree of which is dependent on the thickness of material being traversed, the electron density of the material, and the energy of the photons. As such, attenuation correction is energy and thus radionuclide specific. Correction for attenuation is typically done using the co-acquired CT data and requires a transformation from Hounsfield units (HU) to units of linear attenuation coefficients which is modelled as a bi-linear relationship.^26^ Additional blurring to match the spatial resolution of the SPECT data is also performed prior to the application of CT-derived attenuation correction maps.

Detected scattered photons must also be corrected for, as Compton scattering events can lead to additional photons being detected even though they in fact originated outside the angle of acceptance of the collimator. Scattered photons lead to inferior contrast as well as incorrect quantification, the degree of which is again dependent on the energy of photons emitted from the source, the material through which they traverse, and the selected energy window and collimator being used. Scattered photons are commonly estimated via multiple energy window methods, such as the dual energy window (DEW) or triple energy window (TEW) methods, before subtraction from the primary photopeak data set. An alternative, albeit less common, approach to subtraction of scattered counts is through direct modelling of scatter in the reconstruction algorithm, which allows for preservation of Poisson properties of the projection data.

Additional corrections for camera dead time have become available with vendor supplied quantitative software and are particularly relevant when imaging a high flux of photons, such as post-therapy imaging for dose confirmation where high levels of activity are in the scanner field-of-view (FoV). A further correction that has implications for quantitative accuracy, particularly in small objects, is that of resolution recovery. Vendors now offer resolution recovery for improved quantification and image quality, typically through collimator-modelled point spread function (PSF) convolution techniques. Correction for resolution effects has been an issue and is particularly more pronounced when utilising quantitative SPECT for theranostics where the medium energy collimator is required for typical therapeutic radionuclides, such as ^177^Lu. Partial volume effects are an additional issue leading to poor spatial resolution and are primarily driven by the need for large voxel sizes in SPECT to obtain sufficient counts statistics, leading to insufficient sampling of small structures. This ultimately results in both voxel spill-in and spill-out effects, which typically lead to large underestimates of concentration measured in small structures that approach 3-5 times the system spatial resolution. Correction methods often involve *post hoc* scaling of concentration based on measured object size and prior modelling, or in some cases algorithms are capable of moving counts back into the associated structure with information provided by the hybrid CT.^27^ A unique approach to resolution losses in the theranostics field has recently been proposed which uses the high-resolution diagnostic PET pairing to inform the reconstruction process of the poor resolution post-therapy SPECT data.^28^ Such an approach does not suffer from the typical Gibbs’ artefacts that are common when using PSF methods and achieves a superior resolution to standard SPECT reconstruction, showing promise for future clinical use.

Whilst Monte Carlo methods are being explored for full photon transport modelling and accurate quantitative reconstruction (eg, being utilised by Hermes Medical’s SUV SPECT), most clinical reconstructions are not based on this approach. Various vendor specific software suites are available to quantify SPECT data (eg, Siemens xSPECT QUANT and the GE Q.Metrix algorithm), which rely on accurate camera calibration against the dose calibrator. Vendor neutral options from third party software vendors are also available for reconstruction.

The introduction of quantitative SPECT reconstruction has sparked avid interest in the possible clinical role of standardised uptake value (SUV)-like measures,^29^ which have previously been reserved for PET interpretation only. SPECT SUV has been successfully investigated for a range of diagnostic studies, including bone disease,^30–32^ cardiac amyloidosis,^33^ and [^67^Ga]Ga-citrate imaging of infection.^34^ Quantitative SPECT has also revolutionised the potential of SPECT-based dosimetry that can aid in patient management in theranostics, and is now common practice for certain therapies such as ^177^Lu-DOTATATE for neuroendocrine tumours,^35–37^ [^177^Lu]Lu-PSMA for metastatic prostate cancer,^38–40^ 166Ho microspheres for the treatment of liver cancer,^21^^,^^41^ and [^99m^Tc]Tc-MAA for treatment planning of radioembolisation.^42^ Ongoing research also suggests its feasibility in the use of imaging and dosimetry following targeted alpha therapy based on ^225^Ac emissions^43^ and ^131^I for thyroid cancer,^44^ amongst others.

## Other advances

Other developments in SPECT/CT technology have also been established that have the potential to make a significant clinical impact. One example is the use of whole-body SPECT/CT re-projected planar data to avoid the need for 2D planar whole-body acquisitions which are done routinely for some studies with the frequent addition of 1-2 bed SPECT/CTs over areas of interest. Whole-body SPECT/CT has only become a reality due to developments in both SPECT hardware and software, allowing for faster acquisition times and accurate stitching of adjacent FoVs. The results are 3D whole-body data which can be fully quantitative, leading to PET-like images and displays such as Maximum Intensity Projections (MIPs). However, due to the historic use of 2D whole-body planar data, moving to fully 3D only can be challenging for standard clinical interpretation. The generation of synthetic planar data was originally introduced when transitioning lung scanning from 2D planar to V/Q lung SPECT.^45^ Expansion to common whole-body clinical SPECT studies, such as bone scintigraphy,^31^^,^^46^^,^^47^ shows great promise in revolutionising the way previously 2D data can be interpreted and reported for clinical patient diagnosis and management as all photons from the SPECT acquisition are used for each re-projected planar image. With the introduction of semiconductor digital SPECT and its improved sensitivity and resolution, this may also extend into lower count studies such as diagnostic ^67^Ga for infection imaging.

Another advancing theme in SPECT/CT technology is the role of artificial intelligence (AI). AI is demonstrating significant impact on many areas of medicine and its application in imaging has many benefits. AI for semi-automated segmentation is used routinely in clinical dosimetry and response assessment based on both anatomical and functional data.^48^ The use of automated assessment of total body tumour burden from post-[^177^Lu]Lu-PSMA quantitative SPECT data has been reported based on deep learning semantic segmentation applied to threshold based segmentation^49^ following the demonstration of such measures as a significant biomarker for response to therapy.^38^ Assessment of dose to organs at risk following ^177^Lu therapy through automated segmentation of kidneys from both CT and SPECT input has been reported.^50^ AI has also been proposed as a possible means for correcting for partial volume losses in quantitative ^177^Lu SPECT through deep learning.^51^ Numerous studies have found benefits in AI for SPECT reconstruction^52^ and voxelized dosimetry.^53^^,^^54^ In addition, there is also evidence of deep learning-based methods for attenuation correction in both SPECT and PET, negating the need for any additional CT dose.^55^ An in-depth review of both AI techniques and radiomics applied to SPECT can be found in the recent publication.^56^

## Discussion and conclusion

There is an abundance of new technology, in terms of both hardware and software, becoming mainstream in clinical SPECT/CT. Such progress is both a driving force behind developments in scanning possibilities and an evolving solution being discovered to meet the demand of new radiopharmaceuticals and both diagnostic and therapeutic applications. The introduction of new materials and geometries has seen some of the limitations of SPECT addressed, improving resolution, image quality and quantification, together with new software for improved modelling of photon transport and increased reconstruction speed. However, certain fundamental obstacles still remain, such as the resolution limits and partial volume losses when imaging small structures and the poor efficiency of SPECT due to the collimator design. With further advances in SPECT/CT technology, particularly with drivers such as AI, the following decade of clinical SPECT scanning will no doubt see more discoveries and new investigations addressing these.

We are at a time when clinical SPECT/CT is becoming comparable to PET/CT in terms of image quality of 3D whole-body data and quantitative accuracy, and we must now begin to fully appreciate what this offers in terms of new clinical applications. Such technology will play a role in shaping a changing diagnostic approach, such as the introduction of SPECT-based SUV equivalent measures to guide clinical management, and will support the move towards patient-specific radionuclide therapy planning and an improved understanding of target and threshold doses for optimal treatment outcome.

Looking at the clinical adoption of PET/CT following its introduction in 2001, it took approximately five years for all PET cameras sold to become PET/CT, but the evolution of SPECT to SPECT/CT has taken closer to 20 years. In 2024, it is estimated that approximately 50% of all gamma camera systems are now SPECT/CT, demonstrating widespread evidence of the adoption of the technology. Combined with the rapidly increasing interest and success of theranostics, where SPECT/CT is necessary for imaging the γ emissions that accompany most β^−^ emitters, SPECT/CT technology will continue to evolve to meet our growing demand.
